# JAK Inhibition in Juvenile Idiopathic Arthritis (JIA): Better Understanding of a Promising Therapy for Refractory Cases

**DOI:** 10.3390/jcm12144695

**Published:** 2023-07-14

**Authors:** Isabelle Melki, Marie-Louise Frémond

**Affiliations:** 1 General Paediatrics, Department of Infectious Disease and Internal Medicine, Robert Debré University Hospital, APHP, Nord—Université Paris Cité, F-75020 Paris, France; 2 Paediatrics, Rheumatology and Paediatric Internal Medicine, Children’s Hospital, F-33000 Bordeaux, France; 3 Laboratory of Neurogenetics and Neuroinflammation, Imagine Institute, Université Paris Cité, Inserm UMR 1163, F-75015 Paris, France; 4 Paediatric Haematology-Immunology and Rheumatology Unit, Necker Hospital, APHP, Centre—Université Paris Cité, F-75015 Paris, France

**Keywords:** juvenile idiopathic arthritis, JAK inhibition, interferon, LACC1, STING, SAVI, COPA

## Abstract

Juvenile idiopathic arthritis (JIA) is a heterogeneous group of diseases with probably differential underlying physiopathology. Despite the revolutionary era of biologics, some patients remain difficult to treat because of disease severity, drug adverse events, drug allergy or association with severe comorbidities, i.e., uveitis, interstitial lung disease and macrophagic activation syndrome. Janus Kinase (JAK) inhibitors are small molecules that target JAK/Signal Transducers and Activators of Transcription (STAT) pathways, which could then prevent the activity of several proinflammatory cytokines. They may provide a useful alternative in these cases of JIA or in patients actually affected by Mendelian disorders mimicking JIA, such as type I interferonopathies with joint involvement, and might be the bridge for haematopoietic stem cell transplantation in these disabling conditions. As these treatments may have side effects that should not be ignored, ongoing and further controlled studies are still needed to provide data underlying long-term safety considerations in children and delineate subsets of JIA patients that will benefit from these promising treatments.

## 1. Introduction

Juvenile idiopathic arthritis (JIA) is a heterogeneous group of chronic diseases of unknown origin that affect the joints, with onset before the age of 16 years [1]. Among them, systemic juvenile idiopathic arthritis (sJIA), classified as an autoinflammatory syndrome, and polyarticular JIA (pJIA), including initial polyarticular JIA (affecting five joints or more) or extended oligoarticular JIA (oJIA), are usually the most complicated to treat. The different subtypes of JIA have multifactorial and diverging proposed pathophysiology mechanisms, involving innate and adaptive immune dysfunction, auto-antibody production and immune cell population dysregulation, such as T cells and monocytes [2]. Of note, cytokines such as IL-1ß, IL-6, TNFα, IL-18 and S100 protein have been implicated in (s)JIA pathology [3,4,5].

Targeted therapies have therefore been developed to specifically antagonise a single cytokine with recombinant monoclonal antibodies or recombinant proteins. Among them, biological therapeutics or biologics are purified treatments from large-scale cell cultures, including vaccines, growth factors, immune modulators, and monoclonal antibodies. Etanercept, which is a circulating-TNFα competitive inhibitor to its cell surface receptors, was the first biologic approved for clinical use in 1999 [6]. Tocilizumab (a recombinant antibody against IL-6), Canakinumab (a recombinant monoclonal IL-1ß antibody) and Anakinra (a recombinant IL-1ß receptor antagonist) were subsequently developed and then approved by the European Medicine Agency (EMA) and/or the US Food and Drug Administration (FDA) in sJIA, respectively, in 2011, 2013 and 2018 [7]. Tocilizumab has also been approved by EMA and FDA for pJIA in 2013 [7]. Finally, secukinumab was recently approved by EMA and FDA for enthesitis-associated JIA and psoriatic arthritis-associated JIA.

Since their first approval in the early 2000s, biologics have revolutionised medical care and prognosis for young patients affected by such chronic and disabling conditions. Nevertheless, despite the therapeutical progress over the last two decades, around one-fourth of patients with JIA remain resistant to those treatments and develop chronic disease courses, with adverse events of long-term steroids, joint impairment and disability, especially in pJIA and sJIA [8]. Moreover, associated conditions to JIA, i.e., uveitis, macrophagic activation syndrome (MAS) or lung features, may constitute serious complications and increase therapeutic challenges for these young patients. Thus, there are still unmet needs for novel drugs to be developed for such conditions.

Studies over the past years have revealed that JAK (Janus kinase)/STAT (signal transducer and activator of transcription) signalling pathways are central to the pathogenesis of several immune-mediated inflammatory conditions, as mirrored in the inborn errors of immunity affecting key molecules in these pathways [9]. JAK/STAT are proteins that transduce intracellular signals of numerous ligands, including cytokines (among them, IL-2, IL-6 and IL-10 family members, as long as type I and II interferons -IFN I and IFN II), growth factors and hormones [10,11,12]. JAK inhibitors are small molecules targeting and specifically inhibiting JAK protein families. Initially developed for myeloproliferative diseases and large granular leukaemia, respectively, for somatic *JAK2* and *STAT3* gain of function (GOF) mutations, those treatments have been subsequently approved based on the results of clinical trials in the context of inflammatory or auto-immune diseases [12], e.g., rheumatoid arthritis and psoriasis in adult patients. JAK inhibitors are now an important class of targeted synthetic DMARDs [12] (tsDMARDs) with the goal of preventing joint damage. Moreover, patients with monogenic type I interferonopathies, i.e., Mendelian diseases driven by inappropriate IFN I pathway, have been treated using JAK inhibitors with promising results [13].

Here, we provide a comprehensive review of the use of JAK inhibitors in JIA, encompassing pJIA and sJIA, i.e., Still disease, and rare Mendelian disorders predisposing to juvenile arthritis.

## 2. What Are JAK Inhibitors?

**Figure 1:**
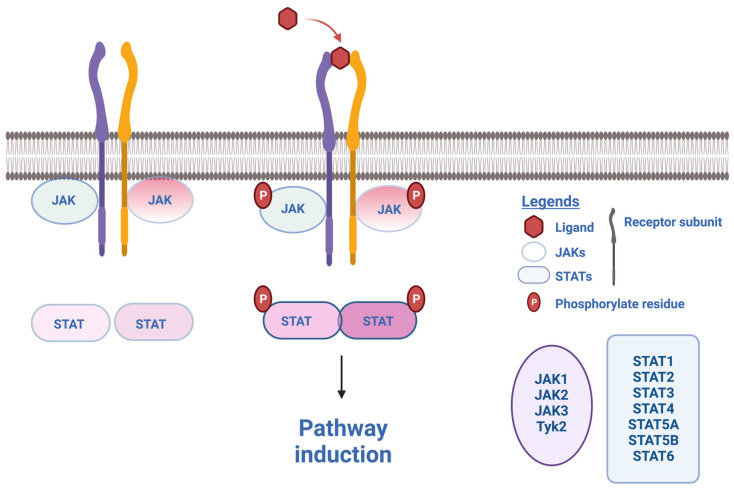
Schematic representation showing JAK-STAT pathways. The ligand (e.g., a cytokine) binds to its specific receptor that recruits two JAK molecules (either the same or two different JAKs), which in turn recruit and activate two STAT molecules through phosphorylation. The STAT complex then translocates into the nucleus and binds to a specific promoter of transcription factors to induce gene expression. JAK: Janus kinase; STAT: signal transducer and activator of transcription. Figure done with Biorender.

The JAK family is constituted by four intracellular tyrosine kinases JAK1, JAK2, JAK3 and Tyk2. JAKs are members of the tyrosine kinase family and represent essential signalling mediators downstream of cytokine receptors. Cytokine binding to its receptor induces phosphorylation of respective JAKs, which then turn into their activated state, subsequently leading to recruitment and activation through phosphorylation of associated STATs (Figure 1). The STAT family includes seven members in mammals: STAT1, STAT2, STAT3, STAT4, STAT5A, STAT5B and STAT6 [14]. After being activated, the STAT complex translocates into the nucleus and induces factor transcription. According to the intracellular interaction between JAKs/Tyk2 and STATs and their specific cytokine receptor, subsequent different downstream signalling will occur. Two types of cytokine families have been individualised. The type I cytokine family includes a total of 29 cytokines, which signal through haemopoietin or type I cytokine receptors, transmembrane molecules that have conserved amino acid motifs (WSXWS), including IL-2, IL-3, IL-4, IL-5, IL-6, IL-7, IL-9, IL-11, IL-12, IL-13, IL-15, IL-21, IL-23, IL-27, IL-31 and IL-35, and also growth hormone (GH), prolactin (PRL), erythropoietin (EPO), thrombopoietin (TPO), G-CSF, GM-CSF, leptin, leukaemia inhibitory factor, oncostatin M (OSM), ciliary neutrophilic factor, cardiotropin-1 (CT1) cardiotropin-like cytokine factor 1 (also referred to as neurotrophin-1 or NNT-1) and thymic stromal lymphopoietin [11]. The type II cytokine family regroups 28 cytokines, which signal through IFN receptors and IL-10 receptors, both of which lack the WSXWS motifs: IFN I, IFN II, IFN III and IL-10 cytokines (IL-10, IL-19, IL-20, IL-22, IL-24 and IL-26) [11].

**Figure 2:**
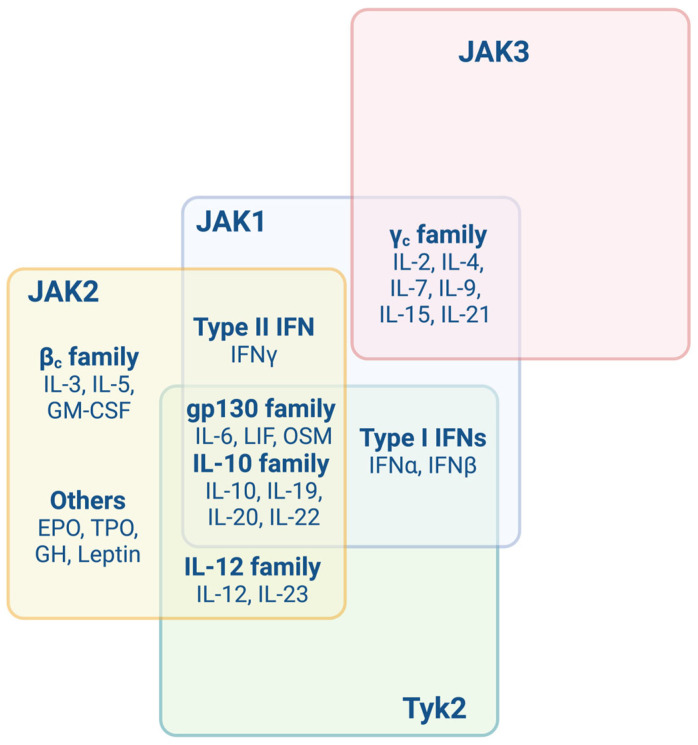
Schematic representation showing signalling pathways through JAKs. EPO: erythropoietin; GM-CSF: granulocyte–macrophage colony-stimulating factor; JAK: Janus kinase; IFN: interferon; IL-: interleukin, LIF: leukaemia inhibitory factor; OSM: oncostatin M; STAT: signal transducer and activator of transcription; TPO: thrombopoietin. Figure done with Biorender.

JAK inhibitors (JAKis) differentiate themselves from biologic DMARDs (bDMARD, i.e., large molecules that must be administered parenterally) as they are orally available. They are referred to tsDMARDs, i.e., small molecules that enter the cytoplasm and directly inhibit kinases or phosphodiesterases. Thus, they affect the regulation of a diverse range of intracellular signalling pathways (Figure 2) [12]. JAKis induce a selective interference in the ATP-binding site of JAKs or prevent phosphorylation of STATs and, hence, downregulate downstream signalling pathways. Thus, their immunomodulatory effect has been used for a broad range of diseases including inflammatory and auto-immune diseases, such as psoriasis or rheumatoid arthritis in adults [15,16,17]. Six JAKis are currently approved for different conditions: ruxolitinib, tofacitinib, baricitinib, peficitinib, upadacitinib and filgotinib (Table 1). They variably inhibit JAK1, JAK2 or JAK3, hence inducing variable downregulation of downstream cytokine effects. For instance, although it was initially thought to selectively block JAK3, tofacitinib variably inhibits JAK1, JAK3 and to a lesser extent, JAK2, both in vitro and in vivo. Tofacitinib was also reported to antagonise, in the same way as baricitinib, the JAK-STAT-mediated differentiation of plasmablasts, T helper 1 (Th1) and T helper 17 (Th17) cells and T cell stimulation by dendritic cells in cell-based assays [12,18,19]. One study showed that filgotinib was more JAK1-inhibitory-specific than upadacitinib, tofacitinib and baricitinib [20] and might, therefore, induce fewer side effects, in particular herpes zoster zona and thrombo-embolic events [21]. Moreover, a pan JAK effect can be expected when using a high dose of JAKis. Nevertheless, future comparison studies are needed to expand our partial knowledge of in vivo downstream-specific JAKi effects. Of note, such effects may partially depend on JAK and STAT isoforms and specific tissue drug penetrance [12].

## 3. JAK Inhibition in Adult Arthritis and Beyond

Initially indicated for myeloproliferative diseases secondary to somatic *JAK2* gain of function mutations, JAKis were then developed for many diseases in adulthood, especially rheumatic diseases, and have been used this last decade. Indeed, JAKis were approved by the FDA and EMA (baricitinib, tofacitinib and upadacitinib, but not filgotinib) for rheumatoid arthritis (RA). They have proved to be at least non-inferior to adalimumab (baricitinib and upadacitinib were more effective, but filgotinib was non-inferior) or abatacept (upadacitinib was superior) [16,22,23,24]. JAK inhibition was also approved by the FDA and EMA for spondyloarthritis (tofacitinib in combination with non-biologic DMARDs and upadacitinib), and phase III trials showed at least similarity to TNF inhibitors [25,26]. In addition, psoriatic arthritis has specific indications for some JAKis (tofacitinib, upadacitinib).

Although inflammatory bowel diseases have distinct pathophysiology from rheumatic diseases, JAK inhibition may target cytokines involved in Crohn’s disease (IFNγ, IL-6 and IL7, with a predominant T_H_1 and T_H_17 cell immune response) and ulcerative colitis (IL-5, IL-13, IL-15 and IL-33 T_H_2 cell associated response). Tofacitinib and upadacitinib were approved for moderate to severe ulcerative colitis after phase III studies and tofacitinib showed efficacy for refractory ulcerative colitis. Studies are currently ongoing for Crohn’s disease, but upadacitinib has already been approved by the FDA in that indication.

Topical ruxolitinib and upadacitinib were approved for atopic dermatitis and non-segmental vitiligo, the latter having shown superiority to dupilumab (monoclonal antibody targeting IL-4 and IL-13), albeit associated with a higher frequency of serious adverse infections, including one fatal course due to influenza [27]. Alopecia areata and palmoplantar pustulosis are other dermatological conditions for which JAK inhibition has proven some efficacy [12,28].

Finally, JAK inhibition is currently being assessed for other autoimmune diseases, such as systemic lupus erythematosous, giant cell arteritis, systemic sclerosis and dermatomyositis [12,29,30].

## 4. JAK Inhibition in Oligoarticular, Polyarticular, Enthesitis-Related and Systemic JIA

Paediatric rheumatology patients differ from adults affected by rheumatic diseases. Indeed, the pathophysiology is usually not specifically the same, especially in genetic conditions, which account for more juvenile forms. Moreover, pharmacokinetics differs in young patients and may need specific future studies. Several case reports and case series paved the way for randomised trials on pJIA and SoJIA, with JAKis used in off-label indications [31,32,33,34,35,36,37,38,39,40,41]. To date, 45 patients have been reported to be at least partially efficiently treated with tofacitinib, 4 pJIA patients with baricitinib and 1 sJIA patient with ruxolitinib (Table A1) [31,32,33,34,35,36,37,38,39,40,41]. Among them, two patients with JIA were efficiently treated during adulthood, respectively, with tofacitinib for microscopic colitis (and arthritis) and with ruxolitinib for EBV-related MAS in previously controlled sJIA [37,41]. Treatment failure has rarely been reported (two non-responders in the single-centre retrospective study by Kostik et al.) [38]. Nevertheless, controlled studies are still needed to confirm if JAKis would constitute promising treatment for refractory JIA or for children who do not tolerate methotrexate (because of hepatitis liver enzymes abnormality or nausea and vomiting) or biologic subcutaneous injections/intravenous infusion.

Table 2 and Table 3 summarise all completed, current and future clinical trials assessing JAKis in the context of JIA. Ruperto et al. were the first to report the results of a double-blind, placebo-controlled, withdrawal phase 3 randomised trial (ClinicalTrials.gov, NCT02592434) on tofacitinib for polyarticular course JIA (extended oJIA, rheumatoid factor (RF) positive or negative pJIA or sJIA without active systemic features) [42]. Between 2016 and 2019, 225 patients aged from 2 years to younger than 18 years were included from 64 PRINTO centres in 14 countries. Among them, 82% had polyarticular course JIA, 9% psoriatic JIA and 9% enthesitis-related arthritis (ERA). Both last groups were included as exploratory endpoints. After the first open-label phase, where all 184 patients with a polyarticular course of JIA received tofacitinib, half of them were assigned to continue tofacitinib, and the rest of them received a placebo. In the tofacitinib arm, more patients achieved the primary endpoint, i.e., lower flare rate (respectively 29% vs. 53%; hazard ratio 0.46, 95% CI 0.27–0.79, *p* = 0.0031) and a significantly longer time to JIA flare than in the placebo group (71% patients remained flare-free in the tofacitinib group). Safety was similar, with mild or moderate adverse events monitored in both groups (77% in the tofacitinib group and 74% in the placebo group). Severe adverse events were reported during the first part of the study in five patients (2%), and three were reported in the placebo group (4%) during the second part. Of note, the authors reported adverse events of special interest in the tofacitinib arm: mild and moderate liver enzyme elevation (respectively, *n* = 2 and *n* = 1), serious infections in three patients (one pneumonia, one epidural empyema and sinusitis in a patient with previous medical history of craniostenosis repair and one appendicitis) and mild monodermatomal non-serious herpes zoster occurred in two patients (1%). No death, malignancies, opportunistic infection, or thrombotic events were observed during the study period.

Thus, only tofacitinib (Xeljanz^®^) is currently approved in the USA by the Food and Drug Administration (FDA) and the European Medicine Agency (EMA) in the context of pJIA in children older than 2 years as a second-line treatment, after failure of methotrexate, along with methotrexate or alone. The recommended FDA/EMA dosages for pJIA and psoriatic JIA are indicated in Table 4. Other studies were conducted (baricitinib for pJIA, with reported efficacy; this study is awaiting publication) or are still ongoing or planned (baricitinib, tofacitinib or upadacitinib for pJIA or sJIA) to complete the initial results obtained in the context of joint features in JIA (Table 4). Nevertheless, JIA patients may develop severe refractory-associated comorbidities, and JAKis may constitute a promising therapeutic approach for these disorders.

## 5. JAK Inhibition in Uveitis, Alopecia Areata, Lung Disease and MAS Associated with JIA

In JIA, uveitis is one of the extra-articular-associated conditions with high morbidity (synechiae, cataract) and risk of blindness in extreme cases if not promptly diagnosed or treated properly [43,44]. TNFα antagonists, such as adalimumab and infliximab, have proven their efficacy in refractory cases upon methotrexate treatment and are currently recommended internationally [45,46]. Nevertheless, some refractory cases are reported upon adalimumab or infliximab treatment, with or without evidence of biologic-specific autoantibodies, and may need alternative treatments. Five cases of JIA-related uveitis efficiently treated with JAKis (baricitinib, *n* = 3; tofacitinib, *n* = 1; upadacitinib, *n* = 1) have been reported to date after failure of several biological treatments including TNFα antagonists [47,48]. One patient treated with upadacitinib achieved clinical joint and ophthalmological remission after tofacitinib failure. Two other patients with JIA-associated uveitis were treated with JAKis, but no information was provided about the treatment efficacy [38]. With the aim of properly answering the question of JAKis efficacy for JIA-related uveitis, an open-label, adalimumab active-controlled, phase 3 clinical multicentre trial (JUVE-BRIGHT) is currently ongoing (Table 3) [49]. Its purpose is to compare adalimumab, a current reference treatment for JIA-uveitis, and baricitinib in children aged 2 to 18 years old. The primary endpoint is the proportion of patients with a response at week 24, and the results may improve treatments offered for JIA-related uveitis.

Of note, three cases of JIA-associated total alopecia areata were reported to be efficiently treated with tofacitinib [38]. The association between alopecia and JIA is scarce, and these patients may have underlying or associated specific auto-immune conditions, which might explain the good efficacy of JAK inhibition in all three cases. In addition, JAKis are approved for alopecia areata in adult patients. Nevertheless, alopecia is not a life-threatening condition, and one might consider infectious risk balance versus the well-being of the child.

A recently reported complication in JIA is drug reaction with eosinophilia and systemic symptoms (DRESS)-like and rapidly progressive interstitial lung disease (ILD) characterised by lymphocytic interstitial inflammation and alveolar proteinosis [2,50]. In addition to being associated with HLA DRB1*15, this severe condition presents with very high levels of IL-18, and one case was reported to be efficiently treated with MAS-825, a drug combining canakinumab and anti-IL-18 [51,52,53]. However, ILD cases have been observed in biologics targeting IL-1ß and IL-6. In addition, it has been hypothesised that IL-1ß inhibition may induce increased levels of IFN I because of a cross-regulation between both cytokines, as observed in subgroups of sJIA patients [54,55]. Such hypersecretion of IFN I could subsequently lead to an IL-18 increase, thus enabling IFNγ-mediated hyperinflammation and paving the way for MAS and ILD [2,56]. Thus, a long-term assessment and, at best, a randomised-controlled trial, would be necessary to become more confident in prescribing this new combined anti-IL1 and IL-18 therapy.

An alternative option would be JAKis, as three cases were separately reported with efficient control of lung disease (tofacitinib, *n* = 2; ruxolitinib, *n* = 1) [38,40,57]. IL-18 is not supposed to be specifically antagonised by JAKis. However, these patients were reported associated with MAS, which is known to be linked to IFNγ, and JAK1/2 inhibition should be efficient to control such a trigger (Figure 2) [52]. Indeed, a specific group of cytokines that signal through the JAK/STAT pathway are the interferons (IFNs), which regroup three families. Type I IFNs (IFN I) mainly include 13 subtypes of IFNα and bind to the specific IFNα receptor (IFNAR) heterodimer (IFNAR1/2), which signals downstream through JAK1 and Tyk2 (Figure 1 and Figure 2). Type II IFN (IFN II) or IFNγ binds to the IFNγ receptor (IFNGR) heterodimer (IFNGR1/2), which signals downstream through JAK1 and JAK2 (Figure 1 and Figure 2) [58]. Type III IFNs (IFN III) include four IFNλ (1–4) and signal through a heterodimeric receptor comprising IL-10 receptor ß and IFNλ receptor 1 (IFNLR1) [59]. IFN I and IFN II are not commonly associated with JIA. Nevertheless, some patient subsets have been reported to harbour elevated levels of IFN I [54,55] and might be included at some point in already individualised or non-classified genetic type I interferonopathies.

## 6. JAK Inhibition in Mendelian Conditions Mimicking JIA

### 6.1. JAK Inhibition in Type I Interferonopathies

Type I interferonopathies are a group of Mendelian auto-inflammatory diseases characterised by constitutive signalling of IFN I [60] and include more than 30 monogenic disorders. Increased production of IFN I or defective retro-regulation drives the constitutive expression of IFN-stimulated genes (ISGs) (also called IFN signature) through the engagement of a common receptor (IFNAR) that subsequently activates JAK1 and Tyk2. The concept of type I interferonopathies was raised in 2011 [61] by Yanick J Crow and supports the hypothesis that the features seen in these syndromes are driven—at least in part—by the excessive or dysregulated IFN I production and should be alleviated using a therapeutic strategy with drugs specifically targeting this pathway [60]. The clinical phenotype of type I interferonopathies extended along with the description of case reports and patient cohorts [60,62]. Of note, it has become clear that a subset of patients present with articular involvement, often with an early onset, a positivity for the RF and a severe presentation [63,64]. Clinical aspects include arthralgia, polyarthritis, Jaccoud-like arthropathy and very few cases of osteonecrosis. In particular, COPA syndrome, due to heterozygous mutations in *COPA*, is associated with joint involvement in around 70% of patients [65]. Interestingly, an ‘isolated’ RF-pJIA can underly such Mendelian interferonopathy, as reported by Bader-Meunier et al. [66]. STING-associated vasculopathy with onset in infancy (SAVI), a severe type I interferonopathy due to gain of function mutations in *STING1*, can also present with joint involvement [64]. However, most of these patients also have at least one of the core features seen in SAVI, i.e., severe skin vasculopathy, systemic inflammation, and interstitial lung disease. Finally, Singleton–Merten syndrome (SMS) is a rare type I interferonopathy caused by heterozygous GOF mutations in *IFIH1* [67], and patients variably present with abnormal calcification of the aorta and cardiac valves, dental caries and early tooth loss, osteoporosis, psoriasis and muscular weakness. Of note, SMS patients can also have Jaccoud-like arthropathy [68], a feature reminiscent of SAVI and COPA syndrome.

In the last 10 years, JAKis have been trialled in type I interferonopathies that are usually poorly responsive to conventional immunosuppressive drugs [13]. When present in these severe conditions, inflammatory joint involvement/arthritis usually responds well to JAKis [64,69,70]. However, the use of JAKis in these rare inherited diseases deserves additional reporting to conclude the efficacy of these drugs on these exceptional ‘JIA’ features. Of interest, a resolution of acro-osteolysis was reported in one SMS patient treated with ruxolitinib [71].

### 6.2. JAK Inhibition in JIA Associated with LACC1 Deficiency

In addition to type I interferonopathies, autosomal recessive mutations in *LACC1* have been described as the first cause of Mendelian JIA [72,73]. *LACC1* encodes the enzyme laccase domain-containing 1, and the recent work by Omarjee et al. suggested that LACC1 deficiency is associated with impaired autophagy in macrophages [74]. This peculiar sJIA or pJIA, with often systemic inflammation, induces progressive joint damage with poor response to aggressive treatment [73]. Two LACC1-deficient patients were treated with baricitinib at the time of publication [74] with partial clinical efficacy (data not published). More cases and/or clinical studies are needed to assess JAKis efficacy and indication in LACC1 deficiency.

## 7. Safety

All new therapeutic strategies should aim for safety (i.e., avoiding drug toxicity and complications, especially infections or malignancies) and efficacy (i.e., control of systemic and joint inflammation, prevention of relapse, avoidance of disease comorbidities and structural damage, normal growth and improving quality of life) on the short and long term.

### 7.1. Infections

The majority of JAKi side effects are infectious. In particular, herpes zoster (VZV reactivation) is the most frequent infection reported in adults treated with these drugs [75,76,77]. This viral reactivation was also reported in children treated with JAKi for different conditions, including JIA [42] and monogenic type I interferonopathies [64,69]. BK virus reactivation was also observed and could progress to severe nephropathy [69]. Other opportunistic or severe infections (toxoplasmosis, tuberculosis, pneumococcal infection) have been documented [69,78], and John Cunningham (JC) virus-mediated progressive multifocal leukoencephalopathy was reported in one adult patient treated with ruxolinitib [79]. It is therefore important to assess infectious diseases before starting JAKi treatment and to monitor replication using repeated polymerase chain reaction (PCR), especially if they are prescribed with other immunosuppressive treatments, such as steroids (Table 5, summarising our daily practice).

### 7.2. Cytopenia

The erythropoietin and thromboietin signalling pathways are affected by JAK2 inhibition, which can lead to cytopenia in a dose-dependent manner [80]. Lymphopenia and a decreased number of NK cells can be observed with tofacitinib, depending on the dose, likely due to the inhibition of JAK3-dependent T-cell functions.

### 7.3. Thombo-Embolic Events

A warning signal for increased risk of thromboembolic complications (i.e., deep vein thrombosis and pulmonary embolism) and major adverse cardiovascular events was raised by post-marketing safety studies on tofacitinib, ruxolitinib and baricitinib, especially in patients carrying other risk factors for such complications (e.g., positivity for antiphospholipid antibodies) [81,82,83]. To our knowledge, these complications have not been reported in children treated with JAKi.

### 7.4. Metabolic Events

Dyslipidemia can be observed upon JAKi treatment as well as weight gain [84,85]. This is likely due to the reduction in leptin signalling through JAK2 inhibition, resulting in hyperphagia contributing to weight gain, as reported in mice [84].

### 7.5. Neoplasic Risk

Type I IFNs are known to be involved in anti-tumoral surveillance. A higher risk of solid tumours or malignant hemopathies was not observed in clinical trials/meta-analyses in adult patients with different immune-related and/or rheumatic diseases treated with JAKi. However, a post-market study on tofacitinib treatment in more than 4000 RA patients (50 years and older) suggested a higher risk of cancer [86]. To our knowledge, these major side effects have not been reported in children treated with JAKi, but post-market studies and surveillance are needed to address this important point.

### 7.6. Others

Shibata et al. reported palmoplantar pustulosis-like eruption upon tofacitinib treatment in one adult patient with pJIA [87].

### 7.7. Drug Interactions

Most of the marketed JAKis are eliminated by metabolism via the cytochrome P450 enzymatic complex, thereby potentially leading to drug–drug interactions, which need to be taken into consideration for multiple drug prescriptions [88]. In contrast, baricitinib and filgotinib are mainly cleared by renal elimination, and drug dosing should be adapted to renal function when using these two JAKis [89].

### 7.8. Withdrawal Syndrome

Discontinuation syndrome was first and mainly reported in patients treated with ruxolitinib for myelofibrosis [90]. It is defined as a life-threatening hyperinflammation syndrome after sudden JAKi interruption, with acute disease symptoms that may occur 24 h to 3 days after drug cessation and may mimic septic shock syndrome [91]. A likely explanation is that ruxolitinib blocks the dephosphorylation and ubiquitin degradation of JAK1 and JAK2, which accumulates and can lead to a notable activation of downstream signalling when ruxolitinib is stopped [92]. Discontinuation syndrome has been also observed with ruxolitinib in the context of monogenic type I interferonopathies [64]. In baricitinib phase 3 trials on RA patients, a brief interruption of baricitinib was associated with a minor increase in RA symptoms [93]. The risk of discontinuation syndrome is likely to vary depending on the underlying condition, the inflammatory status of the patient (i.e., implicated cytokines) and the JAKi being used, but it should not be ignored. This also indicates the need for careful tapering of the drug when JAKi is discontinued.

### 7.9. Long-Term Considerations

The long-term effects of JAKi are currently unknown, especially in children, and this emphasises the need to carefully evaluate the benefit/risk balance before initiating such treatments. Given the wide range of signalling pathways affected by JAKis, concerns about growth, pubertal development and bone metabolism were also raised, particularly in the paediatric population. Indeed, growth hormone signal through JAK2 and the JAK-STAT pathway is also involved in both bone-protective and bone-degrading properties through diverse cytokines. Of note, using mice models, tofacitinib and baricitinib displayed a bone-sparing effect at steady states and in inflammatory conditions [94]. Teratogen risk is also uncertain. Overall, long-term and prospective follow-up studies are required to better assess such complications in adults and children, as well as infectious, neoplastic and thrombo-embolic events. Nevertheless, the assessment of whether patients with JIA should be treated with JAKis has been largely facilitated using the large randomised controlled trials that have been and are being carried out.

## 8. Perspectives

The use of JAKis for almost one decade in human diseases has brought promising therapeutic effects for numerous indications, among them, the field of rheumatology.

Specifically, in paediatric inflammatory arthritis, the emerging use of JAKis has not replaced conventional therapeutic strategies, even if they are administered orally, which is different from biologics. Nevertheless, they provide a useful alternative for some patients with severe, complicated and refractory sJIA, and may be the bridge for haematopoietic stem-cell transplantation (HSCT) in these disabling conditions [95]. Unmet needs remain, in particular (i) the long-term safety of such drugs administered to young developing children, (ii) accurate biomarkers to monitor drug efficacy and (iii) pharmacokinetics and drug-dosage efficacy in children. In the future, it is possible to expect the development of novel targeted drugs, more specifically, inhibiting one JAK or other relevant components of the type I or type II IFN pathway. However, their use in the context of JIA would have to be evaluated since the type I IFN pathway is not the centre of the pathophysiology in these diseases. Beyond IFNs and JAK-STAT pathways, it is possible to envision the expansion of so-called ‘precision medicine’ for patient care with the use of cutting-edge technologies such as single-cell transcriptomics [96].

## Figures and Tables

**Table 1:** Currently approved marketed JAK inhibitors (1 July 2023).

Name	Specificity	Elimination	FDA Approved Indications	EMA Approved Indications
Baricitinib	JAK1, JAK2	Urine excretion	RA, hospitalised adults with COVID-19	RA
Fedratinib	JAK2, FLT3	Metabolism by cytochrome P450 complex	Intermediate-2 or high-risk primary or secondary (post-polycythemia vera or post-essential thrombocythemia) MF	Myeloproliferative disorders, primary MF
Filgotinib	JAK1	Urine excretion	None	RA
Peficitinib	pan-JAK	Metabolism independent of cytochrome P450 complex	None	None
Ruxolitinib	JAK1, JAK2	Metabolism by cytochrome P450 complex	Topical treatment of AD and vitiligo	Topical treatment of vitiligo
Tofacitinib	JAK1, JAK3 (JAK2)	Metabolism by cytochrome P450 complex	RA, SPA, PsoA, moderate to severe UC, pJIA	RA
Upadacitinib	JAK1	Metabolism by cytochrome P450 complex	RA, SPA, PsoA, moderate to severe UC and CD, AD	RA

Abbreviations: AD: atopic dermatitis; CD: Crohn’s disease; COVID-19: coronavirus disease 2019; JAK: Janus kinase; pJIA: with polyarticular course juvenile idiopathic arthritis; FLT3: Fms-like tyrosine kinase 3; MF: myelofibrosis; PsoA: psoriatic arthritis; RA: rheumatoid arthritis; SPA: spondyloarthritis; UC: ulcerative colitis.

**Table 2:** Completed randomised controlled studies on JIA patients treated with JAK inhibitors (April 2023).

Drug	Study (Phase)	Dose	Study Time	Population	N	Age Criteria	Primary Outcome	Main Conclusion
**Tofacitinib (Xeljanz^®^)**	A3921104, NCT02592434 (Phase 3)	2–5 mg BID	44	ERA, pJIA, PsoJIA	225	2–17 years	Disease flare	Effective
**Baricitinib (Olumiant^®^)**	JUVE-BASIS, NCT03773978 (Phase 3)	4 mg (9–18 years) 2 mg (<9 years)	44	pJIA, extended oJIA, ERA, PsoJIA	220	2–18 years	Disease flare	Effective

Abbreviations: ERA, enthesitis-related juvenile idiopathic arthritis; oJIA, oligoarticular juvenile idiopathic arthritis; pJIA, polyarticular juvenile idiopathic arthritis; PsoJIA: psoriatic juvenile idiopathic arthritis; sJIA, systemic juvenile idiopathic arthritis.

**Table 3:** Ongoing, recruiting, and future studies on JIA patients treated with JAK inhibitors (April 2023).

Drug	Study	Sponsor	Population	Region	Study Duration	Primary Outcome
Baricitinib (Olumiant^®^)	JUVE-BALM, NCT04088396	Eli Lilly	sJIA 1–18 years	Global	2/2020–4/2023	Phase 3 Disease flare
Baricitinib (Olumiant^®^)	JUVE-X, NCT03773965	Eli Lilly	JIA (pJIA, oJIA, PsoJIA, ERA, sJIA) 1–18 years	Global	4/2019–12/2030	Phase 3 Long term safety
Baricitinib (Olumiant^®^) vs. Adalimumab	JUVE-BRIGHT, NCT04088409	Eli Lilly	JIA uveitis, chronic anterior ANA+ uveitis	Europe	9/2019–6/2022	Phase 3 Clinical response and safety
Tofacitinib (Xeljanz^®^)	A3921165, NCT03000439	Pfizer	sJIA	Global	2/2018–8/2023	Phase 3 Disease flare
Tofacitinib (Xeljanz^®^)	NCT01500551	Pfizer	JIA 2–18 years	Global	3/2013–11/2025	Phase 3 Long term safety
Tofacitinib (Xeljanz^®^)	NCT05754710	Pfizer	pJIA and PsoJIA 2–18 years	Korea	8/2023–1/2027	Phase 3 Long term safety
Upadacitinib (Rinvoq^®^)	ABT494, NCT03725007	AbbVie	pJIA 2–18 years	Global	6/2019–8/2027	Phase 1 PK, safety, tolerability
Upadacitinib (Rinvoq^®^) vs. Tocilizumab	NCT05609630	AbbVie	sJIA 1–18years	Global	3/2023–12/2028	Phase 3 Clinical response

Abbreviations: ERA, enthesitis-related juvenile idiopathic arthritis; oJIA, oligoarticular juvenile idiopathic arthritis; pJIA, polyarticular juvenile idiopathic arthritis; PK, pharmacokinetics; PsoJIA, psoriatic juvenile idiopathic arthritis; sJIA, systemic juvenile idiopathic arthritis.

**Table 4:** Recommended tofacitinib administration.

Body Weight (BW)	Dosage
10 kg ≤ BW < 20 kg	3.2 mg (3.2 mL oral solution) twice daily
20 kg ≤ BW < 40 kg	4 mg (4 mL oral solution) twice daily
BW ≥ 40 kg	5 mg (one 5 mg tablet or 5 mL oral solution) twice daily

**Table 5:** Practical prescription and follow-up advice.

What to think of before prescribing JAKi		
Infectious contraindication	Carry out a complete clinical examination and the following investigations before the introduction of these treatments to eliminate: An active infection, in particular, viral: viral serologies (HIV, HBV, HCV, VZV) and viral PCR (BK virus blood). These can be monitored after treatment initiation. If features are evocative of an infectious condition: a chest X-ray or bacterial urine analysis may be performed. Tuberculosis: interferon-gamma release assays (IGRAs) or a tuberculin skin test (TST). In patients who have not had chickenpox before JAKi initiation, anti-VZV vaccination can be discussed if it is not contraindicated (e.g., live attenuated vaccine is contraindicated in the event of immunosuppressive treatment). Treatment with JAKi will then only be started one month later.	
Thrombo-embolic risk	This should be looked for using, for example, antiphospholipid test. Dyslipidaemia: complete lipid profile.	
Treatment association	Pay specific attention to associated treatments, especially treatments with cytochrome p450 inhibitors (except filgotinib and baricitinib, see Table 1).	
Kidney function	Adapt dosage of medication to kidney function (filgotinib and baricitinib).	
What to monitor after prescribing JAKi		
Type	Investigation(s)/Management	Frequency
Infectious monitoring	*VZV* In a VZV seronegative patient: short prophylaxis is recommended in the event of suspected contagion. Depending on the level of immunosuppression, prophylaxis with anti-VZV Ig can be discussed. Curative treatment is prescribed in the case of suspected infection or reactivation of VZV (chickenpox or zoster) orally or with IV depending on the severity. *BK virus* JAKi can cause BK virus reactivation, as reported in [69]. Monitor BK viral load in the blood (PCR) as well as kidney function. Clinical follow-up for infectious side effects.	To start on day 7 of suspected contagion for 15 days. Within 96 h after infection. Before initiation of JAKi, at the start of treatment, and then regularly (initially monthly for the first 3 months, then every 3–6 months) Each visit.
Growth and weight gain	Clinical follow-up: height and weight.	Each visit: Before initiation of JAKi, at the start of treatment, and then regularly (initially monthly for the first 3 months, then every 3–6 months).
Haematology follow-up	Laboratory monitoring: red and white blood count.	Before initiation of JAKi, at the start of treatment, and then regularly (initially monthly for the first 3 months, then every 3–6 months).
Liver follow-up	Laboratory monitoring: liver enzymes.	
Lipid profile follow-up	Laboratory monitoring: lipid profile.	

## Appendix A

**Table A1:** JAK inhibitors reported in JIA in the literature before April 2023.

Paper (First Author)	Drug	JIA Subtype	Associated Complications	Number of Patients	Associated Drugs	Efficacy
Zhang [31]	Tofacitinib 2.5 mg BID	sJIA	-	1	Tocilizumab	Yes
Gillard [32]		sJIA	-	2	-	Yes
Pin [33]	Tofacitinib 5 mg BID	pJIA	-	1	Methotrexate	Yes
Rahman [34]	Tofacitinib	Refractory JIA (37% ERA, pJIA RF+, sJIA)		27		Yes
Vukić [35]	Tofacitinib	pJIA	-	1		Yes
Huang [36]	Tofacitinib 2.5 mg BID	sJIA		1		Yes
Tseng [37]	Tofacitinib 5 mg BID	pJIA	Microscopic colitis (at 58 years old)	1		Yes
Kostik [38]	Tofacitinib 0.15–0.5 mg/kg/day	Moderate to severe JIA (9 pJIA 1 oJIA + alopecia 1 ERA 4 sJIA)	Alopecia areata (1/15)	15		CR: 7/15 2 NR (1 pJIA/1 sJIA)
Maccora [39]	Baricitinib 4 mg/day	pJIA		4		Yes
Bader-Meunier [40]	Ruxolitinib 1 mg/kg/day	sJIA	ILD	1		Yes
Macaraeg [41]	Ruxolitinib	sJIA	MAS in adulthood after EBV exposition	1		Yes

Abbreviations: BID: bis in die, twice a day; CR: complete remission; ERA, enthesitis-related juvenile idiopathic arthritis; ILD: interstitial lung disease; MAS: macrophagic activation syndrome: NR: non-responders; oJIA, oligoarticular juvenile idiopathic arthritis; pJIA, polyarticular juvenile idiopathic arthritis; PK, pharmacokinetics; psJIA, psoriatic juvenile idiopathic arthritis; sJIA, systemic juvenile idiopathic arthritis.
